# Dose-Dependent Effects of Intratesticular Adipose-Derived Mesenchymal Stem Cell Injection on Heat-Induced Spermatogenesis Disorder in Wistar Rats: Focus on Apoptosis and Oxidative Stress

**DOI:** 10.30699/ijp.2025.2063503.3477

**Published:** 2025-11-11

**Authors:** Maryam Arbabi Dastgerd, Saeedeh Shojaeepour, Masoud Imani, Reza Bahramnezhad, Mona Saheli, Shahriar Dabiri

**Affiliations:** 1 Department of Basic Sciences, Faculty of Veterinary Medicine, Shahid Bahonar University of Kerman, Kerman, Iran; 2 Department of Clinical Sciences, Faculty of Veterinary Medicine, Shahid Bahonar University of Kerman, Kerman, Iran; 3 Department of Anatomical sciences, Afzalipour Faculty of Medicine, Kerman University of Medical Sciences, Kerman, Iran; 4 Pathology and Stem Cell Research Center, Kerman University of Medical Sciences, Kerman, Iran

**Keywords:** Mesenchymal Stem Cells, Adipose Tissue, Testis, Hyperthermia, Spermatogenesis, Oxidative Stress, Animal Study

## Abstract

**Background & Objective:**

Spermatogenesis is a temperature-dependent process, and testicular heat stress can cause spermatogenic failure by inducing cell apoptosis and oxidative stress, ultimately leading to male infertility. Adipose-derived mesenchymal stem cells (AMSCs) have been considered an effective therapy for various tissue degenerations, demonstrating the ability to stimulate testicular regeneration and restore spermatogenesis. The current study focuses on the therapeutic potential of AMSCs on semen quality, testicular morphological changes, and oxidative stress parameters in rats exposed to heat stress.

**Methods:**

In this experimental study, 35 adult male rats were randomly assigned to five groups: Group I (control), Group II (vehicle), Group III (heat stress group, temperature-humidity index: 43 °C for 20 minutes), and Groups IV and V (treatment groups receiving 0.5×10⁶ and 1×10⁶ AMSCs, respectively, on the second and fifteenth days after heat stress induction). Sixty days after heat stress exposure, the animals were euthanized; serum testosterone levels and oxidative stress biomarkers were analyzed, and the testes and epididymis were collected for histological and sperm evaluation.

**Results:**

Scrotal heat stress caused deleterious effects on testicular histological structure and function. Testosterone levels and total antioxidant capacity were significantly reduced in the heat stress group. The 1×10⁶ AMSCs-treated group showed moderately preserved testicular tissue morphology. Apoptotic spermatogonia and primary spermatocytes decreased significantly in the AMSCs treatment groups in a dose-dependent manner. Malondialdehyde levels and total antioxidant capacity were also improved. Progressive sperm motility, sperm count, and viability were notably enhanced in the AMSCs-treated groups.

**Conclusion:**

A single dose-dependent injection of AMSCs demonstrated regenerative properties that improved with increasing cell number. Overall, administration of 1×10⁶ AMSCs can alleviate testicular damage and promote the spermatogenesis process in testicular hyperthermia.

## Introduction

According to the World Health Organization, about 17.5% of couples suffered from infertility in 2023, with half of these cases attributed to male factors (1). Male infertility occurs for numerous reasons, including long-term exposure to heat, testicular torsion, varicocele, genetic factors, hormonal disorders, malnutrition, certain medications, exposure to heavy metals, and obesity (2). The detrimental effects of heat stress on male mammalian reproductive organs have been well documented and include testicular atrophy, germ cell apoptosis and autophagy, compromised sperm quality, and transient or permanent male sterility (3). The scrotal testes are normally maintained at a temperature 1.5–2 °C below core body temperature (4). Exposure to heat stress during puberty and adulthood can profoundly affect Sertoli cell maturation, Leydig cell activity, and testosterone production (5). In recent years, heightened interest has been shown in the mechanisms and biological effects of testicular heat stress. Heat stress, as a natural hazard, disrupts the blood–testis barrier by interfering with connections between adjacent Sertoli cells (6–8). It also leads to the degradation of endothelial cells within testicular microvessels and promotes inflammatory responses (9). Heat stress triggers the overproduction of reactive oxygen species (ROS), such as hydroxyl radicals (OH⁻), superoxide (O₂⁻), and hydrogen peroxide (H₂O₂) (10). These ROS cause lipid peroxidation and DNA damage. Additionally, heat stress alters mitochondrial membrane permeability, increasing cytosolic cytochrome c levels, which in turn activates caspase-3. This mitochondrial dysfunction and ROS overproduction create oxidative stress, leading to cellular injury, apoptosis, and ultimately spermatogenic failure (11).

Adipose-derived mesenchymal stem cells (AMSCs) are considered a promising therapeutic candidate due to their accessibility, self-renewal capacity, robust ex vivo expansion, and ability to differentiate into various cell types. AMSCs secrete a range of cytokines, growth factors, and angiogenic and immunomodulatory agents (12). In addition to their differentiation potential, they prevent lipid peroxidation by secreting antioxidant substances, thereby enhancing testicular function via paracrine mechanisms (12). AMSCs have been used as potential cellular therapies to ameliorate various human and animal diseases in recent years, owing to their antioxidant, anti-inflammatory, and regenerative properties (13,14). AMSCs have demonstrated protective effects against testicular damage induced by cisplatin, testicular torsion, and varicocele (15–17).

To our knowledge, no existing studies have assessed the therapeutic potential of different doses of intratesticularly injected AMSCs on heat stress–induced testicular injury. This study was designed to investigate the dose-dependent protective effects of AMSCs on germ cell survival, testosterone levels, sperm parameters, and oxidative stress in the testes of rats subjected to heat-induced injury.

## Materials and Methods

The ethical considerations regarding the storage and handling of laboratory animals in this randomized experimental study have received approval and been documented by the Ethics Committee of Kerman University of Medical Sciences, by IR.KMU.AEC.1403.041.

### 1. Experimental design

Thirty five adult male Wistar rats, approximately two months and weighing around 200-230 grams, were obtained from the animal facility of Afzalipour Kerman Medical School, Kerman, Iran. The number of animals used in the experimental study was calculated using the following formula, with a significance level of 0.05 and a power of 80%.

$n={\left(\frac{Z_{\alpha /2}+Z_{\beta }}{d/s}\right)}^{2}$

 The animals were kept in the laboratory animal facility at the Faculty of Veterinary Medicine for one week to acclimate to the environmental conditions. They were maintained under standard conditions with adequate access to water, food, heat (23±1°C), a relative humidity (40±5%) and a regulated 12-hour light/12-hour dark cycles. The animals were randomly assigned to five equal groups, with seven animals in each group: 

Group I (control): Rats were anesthetized on the first day and the distal third of their body was immersed in a water bath at 35 °C temperature for 20 minutes, then they kept in a standard protocol for 9 weeks (18). Group II (vehicle): the distal third of their body was placed in a water bath at 35 °C temperature for 20 minutes on the first day. On the second and fifteenth days, 100 μl of DMEM (Dulbecco's Modified Eagle Medium) as vehicle was injected into their rete testis. Group III (heat stress): the distal third of their body was placed in a water bath at 43 °C temperature for 20 minutes on the first day. Group IV: after induction of heat stress, 0.5×10^6^ passage AMSCs suspended in 100 μl medium was injected into their rete testis on the second and fifteenth days. Group V: after induction of heat stress, 1×10^6^ passage AMSCs suspended in 100 μl medium was injected into their rete testis on the second and fifteenth days. At the outset and termination of the experiment, all animals were weighed.

### 2. Expansion and transplantation of adipose-derived mesenchymal stem cells

Characterized human adipose-derived MSCs at passage 2 were obtained from the Stem Cell Bank of the Anatomy Department at Kerman University of Medical Sciences (19). As previously reported, flowcytometry analysis confirmed mesenchymal stem cells based on their surface markers; the cells expressed CD105 (74.10%), and CD90 (93.24%), and only a negligible percent of the cells exhibited hematopoietic markers, CD34 and CD45. In addition, adipogenic and osteogenic differentiation of MSCs were confirmed by oil-red and alizarin-red staining. Upon thawing, characterized MSCs exhibiting approximately 90% viability were cultured in T75 flasks containing 12 mL of complete medium. The complete medium was composed of DMEM-F12 medium with 1% penicillin/streptomycin and 10% fetal bovine serum. The cell flasks were incubated under 95% humidity and 5% CO_2_ at 37˚C until they reached 80% confluence. Medium were changed every 2-3 days. At transplantation time, the cells at passages 4-6 were detached using Trypsin–EDTA. Animals in groups IV and V received 0.5×10^6^ and 1×10^6 ^AMSCs, respectively.

### 3. Sample collections

After 60 days, the treatment period was end and anesthesia of rats were induced by intramuscular injection of ketamine hydrochloride (90 mg/kg Body Weight) and xylazine hydrochloride (5 mg/kg BW). 4 ml of blood samples were collected from direct insertion of sterile needle (gage 21) into the exposed heart and then it was poured into the sterile tubes free of EDTA, subsequently centrifuged at 3000 rpm for 30 minutes. Serum was carefully isolated and stored at deep freeze (−70 °C) for biochemical and hormone analysis. Ultimately, both the testes and epididymis were excised, weighed and preserved for histopathological examination and sperm analysis respectively.

### 4. Spermiogram test

Sperm samples were obtained from the caudal epididymis and were incubated in alpha MEM medium for 30 minutes in 37°C for release of epididymal sperm storage. Concentration, motility and viability of samples were assessed based on WHO (World Health Organization) recommendations (20). For determination of sperm count, 10 μL of the sperm rich medium was diluted with identical volume of Phosphate buffered saline including 0.02% formaldehyde fixative. Then sperm counted in 10 μL by Neubauer haemocytometer (Precicolor, Germany) and through light microscopy at 400 x. Assessment of live/dead ratio of sperm samples was determined by vital staining. Immediately after remove from the incubator, 10 𝜇l of the sample was incorporated into 10 μL eosin and nigrosine (1% eosin Y and 5% nigrosine). After 30 seconds 20𝜇l of mixture was smeared on a glass slide and examined under an optical microscope (400 x magnification). 200 spermatozoa per slide were randomly counted. Head of live sperm appear white and in the dead sperms coloring pink or red. To assess the motility of sperm samples, 10 𝜇l of the sperm medium was placed on a clean glass slide and overlaid with slip and evaluated at 400x magnification (Olympus BX51, Tokyo, Japan). At least 300 spermatozoa were counted.

### 5. Measurement of Gonadosomatic Index (GSI)

Testicular weights of each rat were recorded and the gonadosomatic index percent (GSI) was obtained by dividing the weight of the testicles by the body weight.

### 6. Serum testosterone and liver enzyme levels assay

Measurement of testosterone and hepatic enzymes, including alanine aminotransferase (ALT) and aspartate aminotransferase (AST) were perfomed in serum samples by an enzyme-linked immunosorbent assay (ELISA) using biovendor kit (BioVendor company, Czech Republic) and Parsazmun kit (PARS AZMUN company, Iran), respectively based on instruction of company.

### 7. Evaluation of Malondialdehyde Measurement

Buege's method with slight changes was used for measurement of MDA (21). In summary, 125 μl of serum was combined with 1.5 ml of phosphoric acid in a tube, and after shaking, 0.5 ml of thiobarbituric acid was incorporated. The test tube was heated in hot water for 45 minutes. Following the addition of 1 ml of n-butanol, the mixture was centrifuged for ten minutes at 100×g. In final step, the pink phase was separated and absorption was determined at 532 nm. The measure of malondialdehyde was derived from the standard curve of tetraethoxypropane.

### 8. Total antioxidant capacity serum levels assay

Ferric-reducing capability of plasma (FRAP) was used for measurement of total antioxidant capacity according to the Benzie and Strain (1996) method (22). The reduction of Ferric-tripyridyltriazine (Fe III-TPTZ) complex to a dense blue-colored ferrous (Fe II) form is done by the plasma at low PH. This complex exhibits a maximum absorbance at 593 nm, and the intensity of the blue color is changed proportion to the antioxidant ability. First, plasma (5 μL) were added to of FRAP reagent (70 μL). Then, the combination was incubated at 37 ℃ for five minutes, and the absorbance was assessed at 593nm. Values of the FRAP were registered as micromolar.

### 9. Testes histopathological study

 At the end of experiment and necropsy of animals, testes were placed in a container containing 10% formalin. Subsequently, the 5-micrometer sections were stained with Hematoxylin and Eosin and histopathological evaluation was conducted by an expert pathologist using a light microscope (Olympus/BX51, Japan) blinded to the group assignments. Five fields per each slide was evaluated. The variables including the diameter of seminiferous tubules, germinal epithelium height, and number of germinal epithelial cell were assessed in about twenty seminiferous tubules per section (23). Johnsen’s score as an indicator of spermatogenesis condition was calculated in each sample (24).

### 10. TUNEL assay

The Terminal deoxynucleotidyl transferase-mediated dUTP nick end labeling (TUNEL) assay was utilized, recognized as a dependable method for identifying and quantifying apoptosis at the single-cell level by labeling DNA strand breaks. For this staining, the in-situ Cell Death Detection Kit, POD (Roche-11684817910 version 15, Germany) was employed. The apoptosis index was determined using the following formula: the average number of total TUNEL-positive spermatogonia and primary spermatocytes divided by the average number of total spermatogonia and primary spermatocytes.

### Statistical evaluation

All statistical evaluation of the obtained data were conducted using SPSS version 26 (Chicago, USA). The Kolmogorov-Smirnov test was used to assess the data distribution and evaluate the normality of the dataset. The values with a normal distribution are presented as the mean ± standard error of the mean. The One-Way ANOVA and Tukey’s tests were employed for the analysis of parametric data, while Kruskal–Wallis test were utilized for non-parametric data. Two-tailed p-values less than 0.05 were deemed to indicate statistical significance**.**

**Table 1:** Effects of heat stress and different doses of AMSCs (0.5 ×10^6 ^and 1×10^6^) on testes gross morphological parameters.

	Control	Vehicle	Heat stress	HS & AMSC 0.5×10^6^	HS & AMSC 1×10^6^
Right testes weight (mg)	122.9±2.1	127.1±2.6	106.9±0.2^ab^	110.1±1.1^ab^	112.5±0.5^ab^
Right testes long diameter (mm)	19.01±0.5	18.3±0.6	14.1±0.25^ab^	15.05±0.3^ab^	16.14±0.34^abc^
Right testes short diameter (mm)	10.3±0.13	10.25±0.24	7.34±0.29^ab^	8.2±0.12^ab^	8.7±0.45^ab^
Left testes weight (mg)	130.2±2.3	135.23±1.8	115.9±7.9^ab^	120.5±0.5^ab^	117.23±1.71^ab^
Left testes long diameter (mm)	20.0±0.05	19.04±0.23	15.19±0.64^ab^	15.9±0.87^ab^	16.5±0.29^ab^
Left testes short diameter (mm)	11.4±0.11	10.7±0.47	8.2±0.17^ab^	8.77±0.25^ab^	8.07±0.78^ab^
Rat weight (gr)	280.9±27.4	283±13.1	275±16.1	275±15.8	280.1±12.5
Gonadosomatic Index	0.09±0.0	0.09±0.01	0.08±0.0	0.08±0.01	0.08±0.0

Results are expressed as mean±SEM. Significant differences (P<0.05) are indicated by ^a^; vs. the control group in the same row, ^b^; vs. the vehicle group in the same row, ^c^; vs. the heat stress group in the same row, ^d^; vs. the HS & cell 0.5×10^6^ group in the same row, ^e^; vs. HS & the cell 1×10^6^ group in the same row.

## Results

### Gonadosomatic Index (GSI) and macroscopic morphological evaluations

No rats died or exhibited any illness during the 9-week study period. There were no statistically significant differences in the body weight of the animals and GSI among all groups (p>0.05). The weights of the right and left testes were significantly reduced in the heat stress (HS), HS+AMSCs 0.5×10^6^ and HS+AMSCs 1×10^6^ groups in comparison to the control and vehicle groups (p<0.05). These parameters increased insignificantly in AMSCs groups compared to the control group (p>0.05). The short and long diameters of both testes were decreased significantly (p<0.05) in the HS, HS+AMSCs 0.5×10^6^ and HS+AMSCs 1×10^6^ groups compared to the other groups (Table 1).

### Spermiogram results

The impacts of HS and HS+AMSCs on the overall epididymal sperm count are presented in Figure 1A. The HS significantly reduced sperm count (2.5 ± 0.11, p=0.000). Intervention of HS with AMSCs could significantly modify the sperm count in a dose-dependent manner compared to the HS group (p<0.01). The sperm count significantly increased in group V compare to group IV.

The survivability of sperm in the HS group was significantly reduced compared to the control and vehicle groups (p=0.000). The percentage of sperm viability in HS+AMSCs 0.5×10^6^ and HS+AMSCs 1×10^6^ groups were dose-dependently increased compared to HS group (p<0.05 and p<0.001 respectively) (Fig.1B).

Figure 1C and 1D illustrates the immotile sperm and sperm progression in the different groups respectively. Sperm progressive motility rate in the HS group was significantly lower (p<0.001) than those of the control and vehicle groups. Injection of AMSCs at different doses could mitigate this effect (p<0.01). The proportion of immotile sperm was reduced in the HS+AMSCs 1×10^6^ group compared to the HS group (p< 0.01) (Fig.1C & 1D).

### Assessment of oxidative stress indicators

**Fig. 2:**
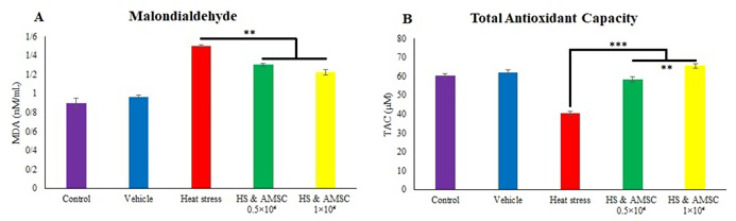
Serum MDA and TAC levels 60 days after heat stress. MDA levels decreased, while TAC levels increased in the treatment groups compared to the heat stress group. Values represent mean±SEM (Error bars) in each group (35=7/group). Significant differences are expressed as * (p˂0.05), **(p˂0.01), and ***(p˂0.001).

The findings from the measurements of oxidative stress biomarkers, including MDA and total antioxidant capacity (TAC) in the serum, are presented in Figure 2. HS significantly elevated (p=0.000) MDA level in the serum. Conversely, treatment by AMSCs resulted in decreased significantly Malondialdehyde levels (p<0.01) compared to the HS group. Furthermore, TAC significantly diminished in the HS group compared to the other groups (p=0.000), whereas AMSCs treatment markedly enhanced TAC levels (p<0.001). TAC in HS+AMSCs 1×10^6^ was significantly higher than HS+AMSCs 0.5×10^6^ group (p<0.01).

### Microscopic results

**Fig. 3:**
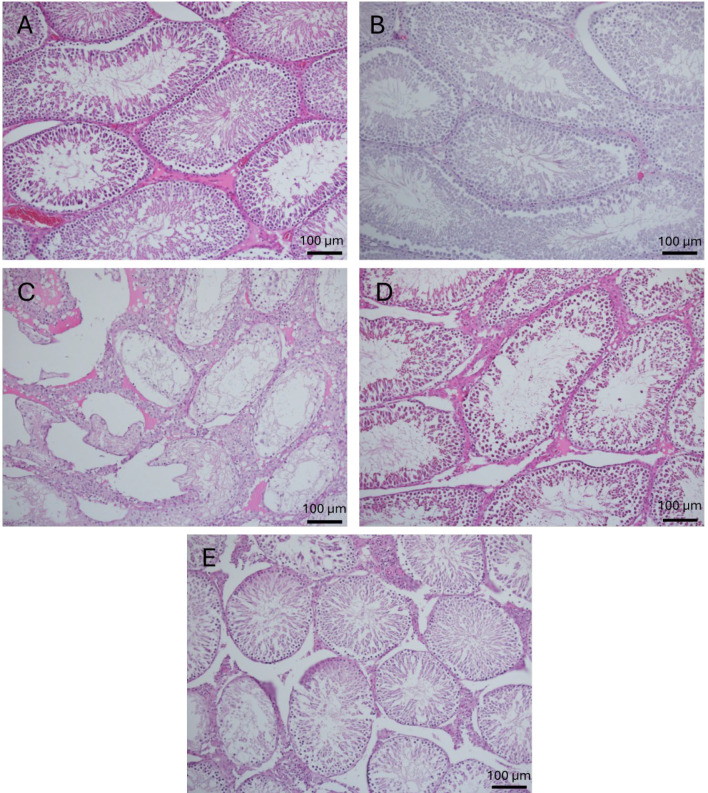
Light micrographs of testicular sections stained with Hematoxylin & Eosin after 60 days (×400). (A) Control & (B) Vehicle groups show the normal looking of testicular tissue and active spermatogenesis in seminiferous tubules. (C) Heat stress group showing absence of the spermatogrnesis and increased number of sertoli cells. Sertoli cells only tubules are seen. (D) Photomicrographs of HS+AMSCs 0.5×10^6^ group showing sloughing and disorganization patterns and (E) in the HS+AMSCs 1×10^6 ^group, almost seminiferous tubules showed normal spermatogenesis along with occasional abnormal maturation of the spermatocytes and Sertoli cell only patterns.

**Fig. 4:**
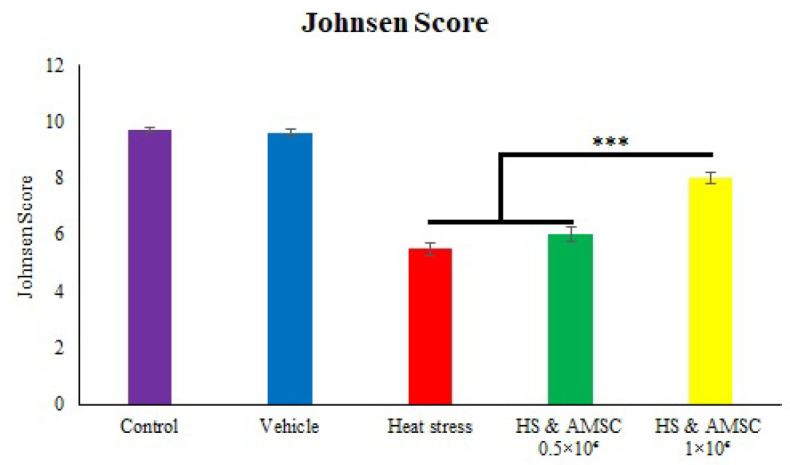
Semi-quantitative indicator of spermatogenesis (Johnson’s score) in testes of different experimental groups. Cell therapy increased germ cells order inside seminiferous tubules in HS+AMSCs 1×10^6^ group. Values represent mean±SEM (Error bars) in each group (35=7/group). Significant differences are expressed as * (p˂0.05), ** (p˂0.01), and *** (p˂0.001).

In control and vehicle groups seminiferous tubules are normal looking with intact basement membrane. Spermatogenesis are normal with presence of the spermatogonia, different spermatocytes, spermatids and sperms at the center with prominent tails. Leydig cells are adequate. In contrast, in the heat stress group, Different damages in the seminiferous tubules are seen as thickened basement membrane, absence of the spermatogenesis and increased number of the Sertoli cells, hyalinization and early fibrosis in the wall of the tubules and interstitial stroma is noted. Mononuclear cell infiltratation was dispersedly seen. Leydig cells are markedly degenerated and decreased in numbers (Figure 3). In the HS+AMSCs 0.5×10^6^ group, histopathological findings showed sloughing and disorganization patterns (maldistribution of the immature spermatocyte cells and sperms). Leydig cells are normal in distribution. However, in the HS+AMSCs 1×10^6 ^group, almost seminiferous tubules (70%) showed normal spermatogenesis along with occasional abnormal maturation of the spermatocytes and Sertoli cell only patterns, too. Leydig cells are hyperplastic. The diameters of the seminiferous tubules and the height of the germinal epithelium in the heat stress group were significantly reduced compared to the control and vehicle groups. While cell treatment insignificantly improved these parameters (Table 2). Figure 4 represents Johnsen’s score and the alterations in the spermatogenic cell line across the different groups.

### Germ cell programmed cell death evaluation

**Fig. 5:**
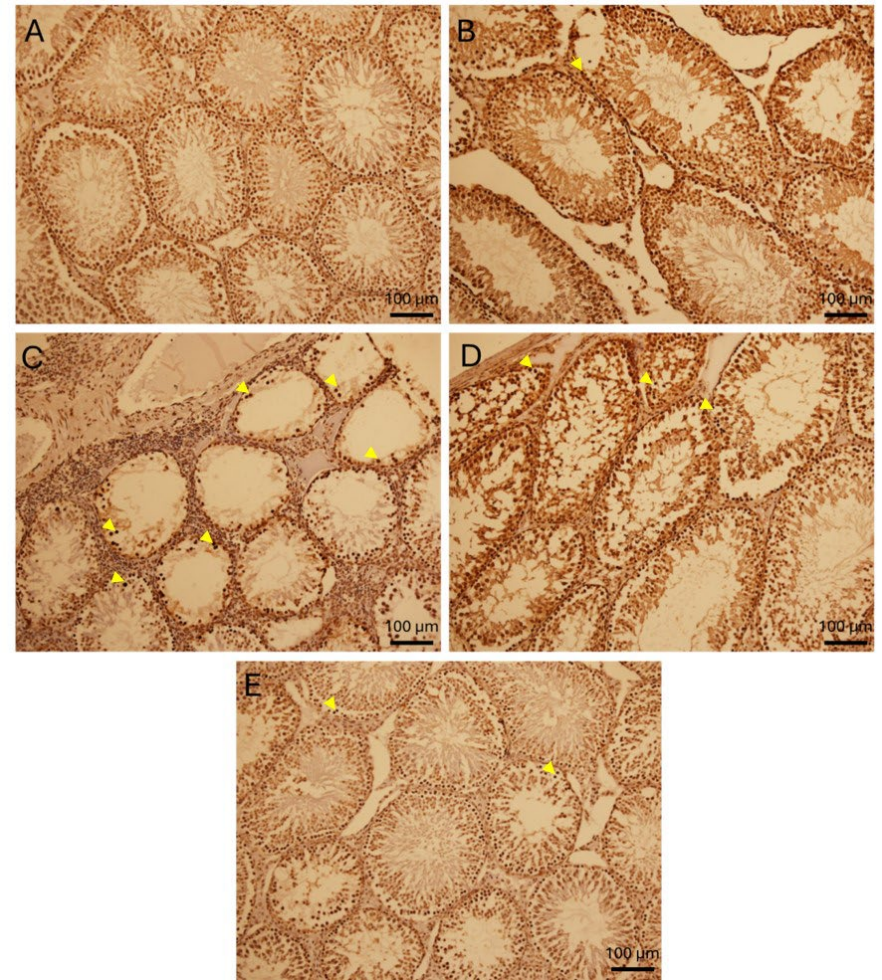
Immunohistochemical staining in rat testis sections after 60 days of heat stress (×400). Photomicrograph of TUNEL-stained rat testicu¬lar sections in control (A) and vehicle (B) groups showed a few numbers (close to zero) of positive spermatogonial cells or primary spermatocytes. The brown cells are broken DNA stained with diaminobenzidine. (C) The most of testicular germ cells is undergoing apoptosis in heat stress group. (D) Marked decrease in apoptotic testicular germ cells was seen in HS+AMSCs 0.5×10^6^ group and (E) HS+AMSCs 1×10^6^ group. TUNEL: terminal dUTP nick-end labeling.

**Fig. 6:**
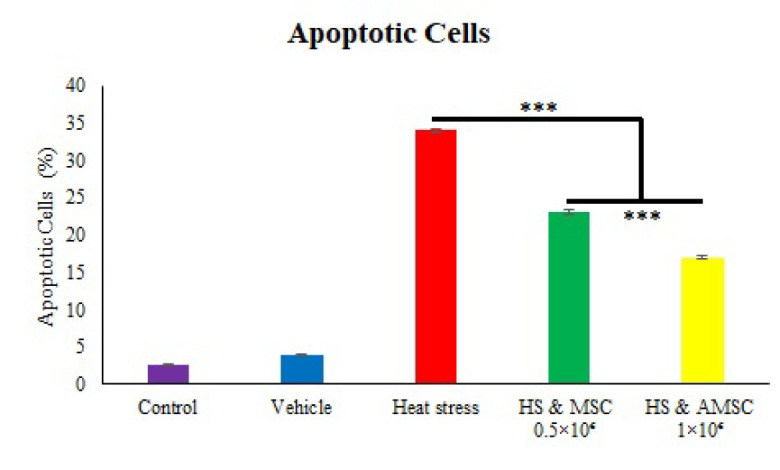
Effect of AMSCs treatment after heat stress on percentage of apoptotic testicular germ cells. Values are expressed as mean ± SEM. Significant differences are expressed as * (p˂0.05), ** (p˂0.01), and *** (p˂0.001).

In this research, apoptotic spermatogonia and primary spermatocyte in rat after heat exposure and injection of AMSCs, were examined by TUNEL labeling (Figure 5). Apoptotic cells in control and vehicle group were visible. TUNEL positive cell markedly elevated in the heat stress group compared to the control and vehicle. AMSCs injection caused a significantly decline in apoptotic cells compared to the heat stress group in a concentration-dependent fashion (p=0.000) (Table 3 & Figure 6). 

**Table 2:** Effects of heat stress and different doses of AMSCs (0.5 ×106 and 1×106) on testicular parameters

	Control	Vehicle	Heat stress	HS & AMSC 0.5×10^6^	HS & AMSC 1×10^6^
Spermatogonia cell number	43.1±2.01	45±1.12	30.2±3.2^ab^	33.5±0.35^ab^	37.5±3.16^bc^
Primary spermatocyte cell number	101±3.9	103±4.07	60.2±2.6^ab^	70.2±1.38^ab^	75±3.1^abc^
Leydig cell number	6±0.12	6±0.02	4±0.33^ab^	5±0.31^ abc^	5.2±0.35^ abc^
Seminiferous tubules diameters (µm)	370.2±4.3	365.2±6.7	250.1±8.9^ab^	261.9±1.29^ab^	280.2±3.54^abc^
Germinal epithelium height (µm)	121.2±5.2	123±9.8	76±4.51^ab^	80.1±7.1^ab^	92.8±3.4^abc^

Results are expressed as mean±SEM. Significant differences (P<0.05) are indicated by ^a^; vs. the control group in the same row, ^b^; vs. the vehicle group in the same row, ^c^; vs. the heat stress group in the same row, ^d^; vs. the HS & cell 0.5×10^6^ group in the same row, ^e^; vs. HS & the cell 1×10^6^ group in the same row.

**Table 3:** Effects of heat stress and different doses of AMSCs (0.5 ×10^6 ^and 1×10^6^) on apoptosis

	Control	Vehicle	Heat stress	HS & AMSC 0.5×10^6^	HS & AMSC 1×10^6^
TUNEL positive SG	1.29±0.01	1.8±0.21	10.5±1.02^ab^	7.25±0.23^abc^	5.±0.02^abc^
TUNEL positive PS	3.03±1.04	4.12±1.07	21±1.6^ab^	16.5±1.01^abc^	14.25±1.5^abc^

Results are expressed as mean±SEM. Significant differences (P<0.05) are indicated by ^a^; vs. the control group in the same row, ^b^; vs. the vehicle group in the same row, ^c^; vs. the heat stress group in the same row, ^d^; vs. the HS & cell 0.5×10^6^ group in the same row, ^e^; vs. HS & the cell 1×10^6^ group in the same row. "SG" (spermatogonia) and "PS" (primary spermatocytes)

### Analysis of serum testosterone and hepatic enzyme activity

A considerable decline was observed in testosterone level in the HS group in comparison with the control and vehicle groups. Testosterone levels increased in the treatment groups compared to the heat stress group, and this increase was significant in the HS+AMSCs 1×10^6 ^group (p<0.05) (Table 4). Assessment of the liver enzymes showed that ALT and AST level did not differ appreciably among groups (p>0.05). (Table 4)

**Table 4:** Effects of heat stress and different doses of MSCs (0.5 ×106 and 1×106) on liver enzymes and testosterone level

	Control	Vehicle	Heat stress	HS & AMSC 0.5×10^6^	HS & AMSC 1×10^6^
Aspartate transferase (AST)	140.1±3.11	142±4.31	150.2±6.2	148.5±2.3	152.1±1.3
Alanine transaminase (ALT)	65.2±4.9	70.1±2.5	60.1±1.7	73.1.±4.1	70.8±4.7
Testosterone	3.75±0.24	3.56±0.22	2.5±0.56^ab^	2.7±0.18^ab^	2.6±0.21^ab^

Results are expressed as mean±SEM. Significant differences (P<0.05) are indicated by ^a^; vs. the control group in the same row, ^b^; vs. the vehicle group in the same row, ^c^; vs. the heat stress group in the same row, ^d^; vs. the HS & cell 0.5×10^6^ group in the same row, ^e^; vs. HS & the cell 1×10^6^ group in the same row.

## Discussion

The outcomes of the current research demonstrated that exposure to heat stress causes marked detriment to spermatogenesis, as validated by results from epididymal sperm analysis and histological assessments of the testes in Wistar rats. Furthermore, the seminiferous tubules of heat stress (HS)–exposed rats exhibited a higher prevalence of apoptotic spermatogonia and a reduction in primary spermatocyte cells. These findings are consistent with previous research indicating the deleterious effects of HS on rat testes, including damage to the seminiferous tubules and reduced testicular size, in conjunction with apoptosis-mediated germ cell loss (18,25,26).

This study showed that the injection of adipose-derived mesenchymal stem cells (ADMSCs) improved spermatogenesis by increasing sperm count and viability, decreasing immotile sperm, and enhancing progressive motility in a dose-dependent manner. In alignment with these results, Tamadon et al. reported that transplanted bone marrow mesenchymal stem cells successfully improved sperm characteristics (27).Our findings also showed significantly decreased testosterone levels and a reduced number of Leydig cells in the HS group compared to the control group. Rizzoto et al. (2020) assessed the effects of acute testicular hyperthermia on serum testosterone levels and testicular gene expression in Nelore bulls, reporting a significant (10-fold) reduction in testicular testosterone and suppression of *STAR*, a gene essential for androgen synthesis, in the hyperthermia group compared to controls (28).

In this study, the number of Leydig cells located adjacent to the seminiferous tubules and total testosterone levels significantly increased in the ADMSC treatment groups. ADMSCs can differentiate into steroidogenic cells expressing Leydig cell markers in animal models and secrete growth factors and cytokines that support endogenous Leydig cell recovery after injury (29). Additionally, recovery of Leydig cell function contributes to improved testosterone production, enhanced spermatogenesis, and better male fertility. However, no significant difference in testosterone levels was observed between the two treatment groups.

In line with earlier findings (30), the results of this study revealed that increased testicular temperature causes disorganization of the seminiferous epithelium and affects all categories of testicular cells, with germ cells being particularly susceptible. Moreover, the level of programmed cell death, assessed via TUNEL staining, was elevated in the spermatogonia and primary spermatocytes of HS-treated rats. In addition to compromising DNA integrity, high temperature exposure reduces the levels of DNA polymerase β, DNA ligase III, and poly (ADP-ribose) polymerase, which play essential roles in DNA repair (6).

ADMSCs treatment notably enhanced the diameters of the seminiferous tubules and the height of the germinal epithelium, and also increased the number of mature spermatocyte cells. Moreover, the number of apoptotic cells significantly decreased in the treatment groups with increasing doses. MSCs upregulate *BCL-2* family anti-apoptotic genes and suppress pro-apoptotic proteins such as *BAX* (31). Moreover, they influence death receptor signaling by modulating the decoy receptor to block extrinsic apoptosis. MSCs mitigate *p53*-mediated apoptosis by modulating its transcriptional activity on pro-apoptotic genes like *NOXA* (32). Nazlı Çil reported that ADMSCs secrete high levels of vascular endothelial growth factor (VEGF), which reduces apoptosis, and insulin-like growth factor-1 (IGF-1), which promotes stem cell proliferation (33).

Testicular oxidative stress, defined by the overproduction of reactive oxygen species (ROS) and impaired function of antioxidant enzymes, has been identified as a primary mechanism underlying HS-induced partial or complete infertility (6,34,35). In the present study, HS significantly enhanced lipid peroxidation and diminished total antioxidant capacity (TAC). Our results, indicating oxidative stress, are consistent with findings reported by other investigators (36–38). Lipid peroxidation, which significantly increased in the HS group, serves as a crucial mechanism of free radical-mediated cellular injury, directly damaging cellular and cytoplasmic membranes. Furthermore, ROS can damage the inner mitochondrial membrane, leading to electron leakage from the electron transport chain and subsequently exacerbating oxidative stress. Defects in mitochondrial function also result in decreased adenosine triphosphate (ATP) availability and reduced sperm motility (39).

Our results demonstrated that ADMSCs treatment enhanced antioxidant power, as evidenced by a notable increase in TAC and a dose-dependent decrease in malondialdehyde (MDA) levels.

Several studies have revealed the ameliorative capacity of adipose-derived mesenchymal stem cells in gonads under oxidative stress conditions (15,19,40,41). The hypothesis is that the paracrine and regenerative activities of ADMSCs assist cells in protecting against injury from free radicals and reducing the harmful effects of heat stress. MSCs enhance cellular antioxidant capacity by promoting stress-induced nuclear translocation of Nrf2 and triggering the expression of antioxidant enzymes such as superoxide dismutase (SOD) and catalase (CAT). Moreover, MSCs improve mitochondrial efficiency through mechanisms such as mitochondrial biogenesis, thereby reducing oxidative stress (42).

This research represents the first study to assess the effects of multiple doses of ADMSCs in HS-exposed rats. Our findings revealed that in both ADMSCs+HS groups, ADMSCs could mitigate the detrimental impacts of hyperthermia on lipid peroxidation (LPO) and TAC in the serum.

ADMSCs, as self-renewable and multipotent cells, are among the most valuable stem cells used in cell-based therapies. Their paracrine and regenerative activities have been demonstrated in various tissues, from animal to human models (19,43,44). ADMSCs secrete a variety of paracrine factors, including VEGF, fibroblast growth factor 2 (FGF2), IGF-1, and interleukin-6 (IL-6). VEGF and FGF2 are crucial for promoting vascularization in damaged tissues, thereby enhancing blood supply and facilitating healing. Additionally, IGF-1 supports both vascularization and tissue regeneration (45).

The antioxidant properties of ADMSCs are attributed to mitochondrial donation, scavenging of reactive oxygen species (ROS) such as hydrogen peroxide and superoxide, and enhancement of the antioxidant defense mechanisms (46,47). Our experiment showed that the 1×10⁶ AMSCs group significantly reduced MDA levels and increased TAC compared to the heat stress group.

In this research, administration of 0.5×10⁶ and 1×10⁶ ADMSCs was able to modulate HS-induced dysregulation of cellular antioxidant enzymes and consequently ameliorate sperm and testicular damage. Ismail et al. (2023) reported that a single injection of 1×10⁶ adipose-derived mesenchymal stem cells in cisplatin-treated infertile rabbits enhanced sperm count and improved testicular function by increasing testicular glutathione (GSH) levels and reducing MDA levels (15). Moreover, Chen et al. (2021) found that treatment with adipose-derived mesenchymal stem cells alleviated the expression of oxidative stress-related proteins such as NOX-1 and NOX-2 and reduced apoptotic and mitochondrial damage markers in rats with testicular torsion-induced ischemia-reperfusion injury (41).

This study also revealed that liver enzyme levels did not differ significantly between groups. No adverse effects on liver function were observed following intratesticular ADMSCs injection.

## Conclusion

The outcomes of this research indicate that administering multiple doses of adipose-derived mesenchymal stem cells, particularly the AMSCs 1×10^6^ group, could promote testicular regeneration, enhance sperm characteristics and restore MDA levels and total antioxidant capacity in heat stress exposed animals. While the precise mechanism of action of AMSCs in cells remains unclear, it may be partially attributed to their antioxidant and anti-apoptotic properties. It is recommended that future studies investigate inflammatory factors and the expression levels of genes related to oxidative stress and apoptosis to ensure AMSCs can be used with greater confidence in clinical trials.

## List of abbreviation

AMSCs: Adipose-derived mesenchymal stem cells 

DMEM: Dulbecco's Modified Eagle Medium

EDTA: Ethylene Diamine Tetra Acetic acid

WHO: World Health Organization

GSI: Gonadosomatic Index 

ALT: Alanine Aminotransferase

AST: Aspartate aminotransferase 

ELISA: Enzyme-linked Immunosorbent assay

MDA: Malondialdehyde

FRAP: Ferric-reducing capability of plasma

TPTZ: 2,4,6-Tris(2-pyridyl)-s-triazine 

TUNEL: The Terminal deoxynucleotidyl transferase-mediated dUTP nick end labeling 

ANOVA: Analysis of Variance

HS: Heat Stress

TAC: Total Antioxidant Capacity 

SG: Spermatogonia 

PS: Primary Spermatocytes

DNA: Deoxyribonucleic Acid 

BCL-2: B-cell lymphoma 2

ATP: Adenosine Triphosphate

SOD: Superoxide Dismutase

CAT: Catalase 

LPO: lipid peroxidation 

VEGF: Vascular Endothelial Growth Factor 

FGF2: Fibroblast Growth Factor 2

IGF-1: Insulin-like Growth Factor 1

IL-6: Interleukin-6

## Data Availability

The data supporting the results of this study are available upon request from the corresponding author.
